# 542. Bromelain Based Enzymatic Debridement: A Retrospective Review of Patient Outcomes

**DOI:** 10.1093/jbcr/irag033.239

**Published:** 2026-04-06

**Authors:** Queenie Dias, Daniela Requena, Matthew D Supple, Sean Hickey, Jonathan S Friedstat, John T Schulz, Jeremy Goverman

**Affiliations:** Massachusetts General Hospital, Boston, MA; Massachusetts General Hospital, Boston, MA; Massachusetts General Hospital, North Reading, MA; University of California San Diego, San Diego, CA; Massachusetts General Hospital, Boston, MA; Massachusetts General Hospital, Boston, MA; Massachusetts General Hospital, Brookline, MA

## Abstract

**Introduction:**

Bromelain based Enzymatic Debridement (BBED) for burn injuries has been practiced for more than 10 years in Europe. Exposure to BBED in the US is still relatively small, as it was only recently approved by the US FDA in December of 2022. We reviewed our initial post-FDA approval experience using BBED for deep partial and full thickness burns.

**Methods:**

An IRB approved, single-center, retrospective study was performed from October 2023 to August 2025. Data included patient demographics, total body surface area (TBSA) burn, enzymatically treated area, mechanism of burn, time post-burn until BBED application, pain management, post-BBED dressings, need for and timing of skin grafting, overall time until complete wound closure, and reconstructive procedures. Wound beds post-BBED were evaluated by experienced burn surgeon and subsequently assessed for the need for surgical closure. Wound beds were also documented by photography.

**Results:**

In our review, 32 Patients (21 Men, 11 Females), age range 18-95 yrs (mean - 46.25 yrs) underwent BBED for 23 flame burns, and 9 scalds. The mean interval from time of burn injury to BBED was 48 hours. Upper extremity (25) was the most common site followed by lower extremity (9), torso (2) and face (1). Mean TBSA was 9% (range 1%-90%) while mean enzymatic area was 5% (1%- 30%). Nine (28.2%) patients healed without skin grafting. Skin grafting was performed in 23 (71.8%) patients. The most commonly used dressings were allografts and xenografts. Time until 95% wound closure averaged 15 days. Two patients required contracture release and grafting, dorsal hand and elbow flexure contracture. No patients required their procedures to be aborted for pain or other adverse effects. All treatments resulted in >95% eschar removal after 1 treatment.

**Conclusions:**

At our burn center, BBED was most commonly used for deep partial and full thickness burns to the upper extremities, including the hands. Pain was well controlled and the procedure was well tolerated. Burns expected to heal without grafting were treated with xenografts and silver foam dressings.

**Applicability of Research to Practice:**

BBED by its virtue of selective eschar removal can help preserve viable dermis and avoid the need for skin grafting in some mixed partial thickness and full thickness burns. In patients who require skin grafting thinner grafts may be harvested.

**Funding for the study:**

N/A.

